# Mitochondrial DNA copy number and heteroplasmy load correlate with skeletal muscle oxidative capacity by P31 MR spectroscopy

**DOI:** 10.1111/acel.13487

**Published:** 2021-10-06

**Authors:** Qu Tian, Ann Zenobia Moore, Richard Oppong, Jun Ding, Marta Zampino, Kenneth W. Fishbein, Richard G. Spencer, Luigi Ferrucci

**Affiliations:** ^1^ Translational Gerontology Branch National Institute on Aging Baltimore Maryland USA; ^2^ Laboratory of Clinical Investigation National Institute on Aging Baltimore Maryland USA

**Keywords:** aging, mitochondrial DNA, skeletal muscle

## Abstract

The association between blood‐based estimates of mitochondrial DNA parameters, mitochondrial DNA copy number (mtDNA‐CN) and heteroplasmy load, with skeletal muscle bioenergetic capacity was evaluated in 230 participants of the Baltimore Longitudinal Study of Aging (mean age:74.7 years, 53% women). Participants in the study sample had concurrent data on muscle oxidative capacity (τ_PCr_) assessed by ^31^P magnetic resonance spectroscopy, and mitochondrial DNA parameters estimated from whole‐genome sequencing data. In multivariable linear regression models, adjusted for age, sex, extent of phosphocreatine (PCr) depletion, autosomal sequencing coverage, white blood cell total, and differential count, as well as platelet count, mtDNA‐CN and heteroplasmy load were not significantly associated with τ_PCr_ (both *p *> 0.05). However, in models evaluating whether the association between mtDNA‐CN and τ_PCr_ varied by heteroplasmy load, there was a significant interaction between mtDNA‐CN and heteroplasmy load (*p *= 0.037). In stratified analysis, higher mtDNA‐CN was significantly associated with lower τ_PCr_ among participants with high heteroplasmy load (*n* = 84, β (SE) = −0.236 (0.115), *p*‐value = 0.044), but not in those with low heteroplasmy load (*n* = 146, β (SE) = 0.046 (0.119), *p*‐value = 0.702). Taken together, mtDNA‐CN and heteroplasmy load provide information on muscle bioenergetics. Thus, mitochondrial DNA parameters may be considered proxy measures of mitochondrial function that can be used in large epidemiological studies, especially when comparing subgroups.

## Graphical abstract

Mitochondrial DNA copy number and heteroplasmy load provide complementary information on mitochondrial oxidative capacity measured in skeletal muscle. The relationship between mitochondrial DNA copy number and muscle bioenergetics is different according to levels of heteroplasmy load. Assessing mitochondrial DNA copy number and heteroplasmy load may provide cost‐effective and accessible insight into muscle mitochondrial function in large epidemiological studies.

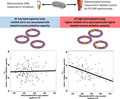

## 1 INTRODUCTION

Mitochondria produce energy for metabolic and functional activity through aerobic metabolism and are also linked to a broad range of cellular processes including apoptosis, and immune signaling, iron and calcium homeostasis, and reactive oxygen species signaling (Gonzalez‐Freire et al., 2015). Dysfunction of these activities has been implicated in the development of chronic disease and is also considered a hallmark of aging (Lopez‐Otin et al., 2013). Muscle is an energetically demanding tissue that is central to decline in physical function in later life. However, commonly used methods to assess mitochondrial function in skeletal muscle, such as respirometry in muscle biopsies and ^31^P magnetic resonance spectroscopy (MRS), are resource‐intensive and impractical for population‐based studies (Coen et al., 2013; Conley et al., 2000; Short et al., 2005). Recent studies have shown that measurements of mitochondrial oxidative capacity in human skeletal muscle via ^31^P‐MRS are associated with multiple chronic diseases and morbidity (AlGhatrif et al., 2017; Brown et al., 2019; Zampino et al., 2020). Techniques have also been developed to estimate mitochondrial DNA copy number (mtDNA‐CN) and heteroplasmy load from whole‐genome sequencing (WGS), most often in blood samples, such as buffy coat specimens (Ding et al., 2015; Qian et al., 2017). However, the relationship between mtDNA‐CN and heteroplasmy load estimated from WGS and mitochondrial function in skeletal muscle is unknown, as mitochondrial characteristics, number, and volume vary by organ, tissue, and cell types. If such a relationship was established, mtDNA‐CN and heteroplasmy load would be invaluable for clinical research, including studies of aging, as proxies for muscle bioenergetic status. In addition, they would form therapeutic targets for drugs and interventions designed to address chronic disease and improve function in aging through improved mitochondrial function (Andreux et al., 2019).

Using data collected from 230 participants of the Baltimore Longitudinal Study of Aging (BLSA), a study of community‐dwelling individuals, we tested the hypothesis that mtDNA‐CN and heteroplasmy load estimated from WGS would be associated with muscle bioenergetic status as assessed with the phosphocreatine (PCr) exponential recovery time constant (τ_PCr_) determined by ^31^P‐MRS (Coen et al., 2013). The mean age of the study sample was 74.7 years, and 53% of participants were women (Table 1). Study participants were free of dismobility, mean usual gait speed of 1.20 m/s with a minimal value of 0.63 m/s (Table 1) (Cummings et al., 2014). In this sample, higher values of τ_PCr_ were associated with older age, indicating age‐related decline in mitochondrial function (Figure S1). BLSA protocols are approved by the National Institutes of Health Institutional Review Board, and all participants provided written informed consent.

**TABLE 1:** Participant characteristics

			Heteroplasmy load ≤3		Heteroplasmy load >3	
	Overall (*n* = 230)		High mtDNA‐CN (*n* = 71)	Low mtDNA‐CN (*n* = 75)	High mtDNA‐CN (*n* = 44)	Low mtDNA‐CN (*n* = 40)
	Mean ± standard deviation or *N* (%)	range	Mean ± standard deviation or *N* (%)	Mean ± standard deviation or *N* (%)	Mean ± standard deviation or *N* (%)	Mean ± standard deviation or *N* (%)
Age, years	74.7 ± 9.8	50 – 93	71.3 ± 9.1	75.2 ± 9.9	75.5 ± 9.8	78.7 ± 9.6
Women	121 (53)	‐	53 (75)	27 (36)	27 (61)	14 (35)
Body mass index, kg/m^2^	26.2 ± 4.3	17.1–42.9	26.6 ± 4.1	26.9 ± 4.9	24.8 ± 3.8	25.9 ± 3.5
6‐meter usual gait speed, m/s	1.20 ± 0.20	0.63–1.81	1.23 ± 0.19	1.20 ± 0.19	1.21 ± 0.23	1.15 ± 0.21
Any cancer	97 (42)	‐	27 (39)	37 (49)	13 (30)	20 (53)
Non‐skin cancer	26 (11)	‐	4 (6)	9 (12)	7 (16)	6 (15)
Diabetes	11 (5)		2 (3)	5 (7)	2 (5)	2 (5)
Current or former smokers	6 (3)	‐	3 (4)	1 (1)	1 (2)	1 (3)
High physical activity category (≥150 min/week)	63 (27)	‐	21 (30)	18 (24)	14 (32)	10 (25)
τ_PCr_	49.4 ± 10.4	22.6 – 3.8	49.4 ± 10.0	48.8 ± 10.1	46.2 ± 10.6	54.1 ± 10.2
PCr depletion, %	59.3 ± 10.2	33.1–91.0	59 ± 10	59 ± 11	59 ± 10	59 ± 10
mtDNA‐CN	219 ± 42	127–366	250 ± 29	188 ± 18	257 ± 34	181 ± 23
Heteroplasmy load	Median (IQR)	range	Median (IQR)	Median (IQR)	Median (IQR)	Median (IQR)
	3 (2)	1–96	2 (1)	2 (1)	5 (2)	5 (3)

mtDNA‐CN was estimated using the mitoCalc algorithm ((Ding et al., 2015; Qian et al., 2017) in WGS data from buffy coat samples. Heteroplasmic variants (mtDNA variants with more than one allele at a DNA site) were identified in the same sequencing data using the mitoCaller algorithm. Heteroplasmy load is represented by the total number of heteroplasmic variants in each individual. In vivo ^31^P‐MRS measurements of the concentrations of phosphorus‐containing metabolites including phosphocreatine (PCr) were obtained from the vastus lateralis muscle using ^31^P MRS at 3T, following a standardized protocol (Choi et al., 2016). τ_PCr_, the PCr exponential recovery time constant measured in seconds, was calculated by fitting time‐dependent changes in PCr peak area to the monoexponential recovery function:

$$PCr\left(t\right)=PCr\left(0\right)+\Delta PCr\times \left(1‐\exp\left(‐\frac{t}{\mathrm{PCr}}\right)\right)$$

where PCr(0) is the end‐of‐exercise PCr signal area and ΔPCr is the decrease in signal area from its pre‐exercise value (Choi et al., 2016). Higher values of τ_PCr_ indicate longer recovery and lower oxidative capacity. Associations of mtDNA‐CN and heteroplasmy load with τ_PCr_ were tested using multivariable linear regression (SAS v9.4; SAS Institute, Inc). After adjustment for age, sex, extent of PCr depletion, autosomal sequencing coverage, white blood cell total, and differential count, as well as platelet count, mtDNA‐CN and heteroplasmy load were not significantly associated with τ_PCr_ (Table S1, Model 1, Model 2).

**FIGURE 1:**
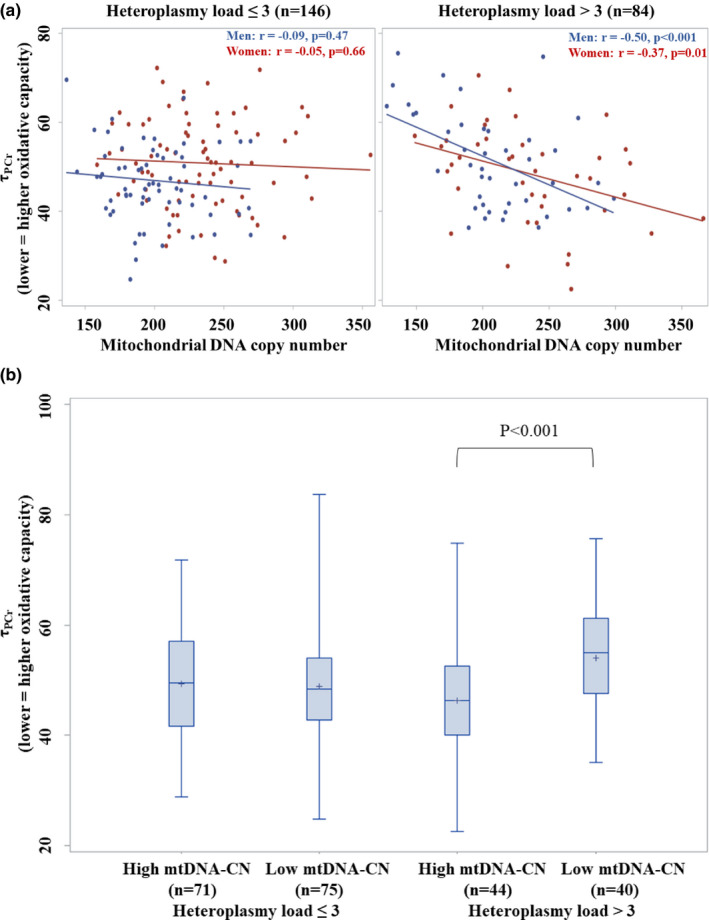
Scatter plots (A) (red: women; blue: men) and boxplots (B) for the unadjusted relationship between mitochondrial DNA copy number and τ_PCr_ in participants with low and high heteroplasmy load. Caption: τ_PCr_, the PCr exponential recovery time constant measured in seconds, higher values of which indicate longer recovery time and lower oxidative capacity. In scatter plots, the specific amount of mitochondrial DNA copy number attributed to low and high heteroplasmy load ranged from 136 to 356, and from 127 to 366, respectively. In boxplots, mitochondrial DNA copy number was categorized by a median split at 216

Since recent data suggest that mtDNA‐CN and heteroplasmy load provide complementary information on mitochondrial function in patients with peripheral artery disease (Gonzalez‐Freire et al., 2020), we also tested the hypothesis that the association between mtDNA‐CN and τ_PCr_ would be different according to levels of heteroplasmy load through the evaluation of an interaction term in multivariable models. There was a significant interaction between mtDNA‐CN and heteroplasmy load (Table S1, Model 3); after stratifying by a median split of heteroplasmy load of 3, higher mtDNA‐CN was significantly associated with lower τ_PCr_ in participants with high heteroplasmy load (Table S2; Figure 1), while there was not a significant association between mtDNA‐CN and τ_PCr_ in those with low heteroplasmy load (Table S2; Figure 1). At high heteroplasmy load, there was also a relationship between mtDNA‐CN and/or τ_PCr_ and gait speed (*p *= 0.06 and <0.001, respectively). Results were not substantially changed after excluding participants with diabetes and non‐skin cancer after full adjustment (β (SE), *p*‐value for those with high (*n* = 68) and low (*n* = 126) heteroplasmy load: −0.315 (0.128), 0.017; and 0.117 (0.113), 0.302, respectively). Results remained similar in sex‐stratified analysis (Table S3).

In this sample of community‐dwelling older adults, we demonstrate that mtDNA‐CN and heteroplasmy load provide complementary information on mitochondrial oxidative capacity measured in skeletal muscle. The significant interaction between mtDNA‐CN and heteroplasmy load indicated that the relationship of mtDNA‐CN with muscle bioenergetics was different according to levels of heteroplasmy load. In particular, we found that mtDNA‐CN was associated with skeletal muscle oxidative capacity only in individuals with high heteroplasmy load. While the mechanism for this interaction remains unclear, we have previously demonstrated that mtDNA‐CN can be associated with both positive and negative health outcomes, for example in participants with and without diabetes (Moore et al., 2018). Our results support the notion that mtDNA‐CN can be assessed at the population level and it can be correlated with other parameters and used to compare subgroups. It may also serve as a measure sensitive to mitochondrial mass in skeletal muscle. However, if higher heteroplasmy load leads to altered mitochondrial damage, increased mtDNA‐CN may indicate a homeostatic response toward increasing mitochondrial biogenesis (Filograna et al., 2020; Qian & Van Houten, 2010). It is also possible that increased mtDNA‐CN may be observed in conjunction with a high level of heteroplasmy load within the context of damage induced by oxidative stress, a strong correlate of impaired mitochondrial function. Thus, increased mtDNA‐CN may reflect the spilling of mtDNA from damaged mitochondria, and in this case, mitochondrial mass may not correlate with function. It can be hypothesized that reduced ATP production may be sensed by AMP kinase which in turn stimulates mitochondrial biogenesis through PGC‐1 alpha leading to increased mtDNA synthesis through transcription factor A, which is required for maintenance of normal levels of mtDNA (Kasashima et al., 2012). The additional mtDNA that is not incorporated in functioning mitochondria may enter the circulation. These data suggest that mtDNA‐CN and heteroplasmy load should be used in conjunction to obtain insight into mitochondrial function.

Our study has certain limitations, including a modest sample size; the BLSA population from which the study sample is drawn is healthier, less diverse, and more well‐educated than the general population. Future studies with larger samples encompassing a greater range of functional status would provide validation of our results. In addition, our study has several strengths. The study sample is comprised of well‐characterized community‐dwelling older men and women, allowing us to control for multiple known covariates: Our observations were robust to adjustment for differential white blood cell and platelet count as well as to exclusion of participants with diabetes and non‐skin cancer, both of which are known to affect mtDNA‐CN.

In sum, when taken together mtDNA parameters, mitochondrial DNA copy number and heteroplasmy load provide an indication of muscle bioenergetics. Assessing mitochondrial DNA copy number and heteroplasmy load may provide cost‐effective and accessible insight into muscle mitochondrial function in large epidemiological studies.

## CONFLICT OF INTEREST

None declared.

## AUTHOR CONTRIBUTIONS

QT developed the study concept and statistical analysis strategy, analyzed, interpreted, and drafted the manuscript. AZM developed the study concept and statistical analysis strategy, interpreted, and reviewed the manuscript. JD, RO, and MP involved in data collection and reviewed the manuscript. KWF and RGS involved in P31 MR Spectroscopy study protocol and reviewed the manuscript. LF designed the study and reviewed the manuscript. All authors edited and approved the manuscript.

## Supporting information

Supplementary Material

## Data Availability

Upon request from the authors.

## References

[acel13487-bib-0001] AlGhatrif, M. , Zane, A. , Oberdier, M. , Canepa, M. , Studenski, S. , Simonsick, E. , Spencer, R. G. , Fishbein, K. , Reiter, D. , Lakatta, E. G. , McDermott, M. M. , & Ferrucci, L. (2017). Lower Mitochondrial energy production of the thigh muscles in patients with low‐normal ankle‐brachial index. Journal of the American Heart Association, 6(9), e006604. 10.1161/JAHA.117.006604 28855165 PMC5634302

[acel13487-bib-0002] Andreux, P. A. , Blanco‐Bose, W. , Ryu, D. , Burdet, F. , Ibberson, M. , Aebischer, P. , Auwerx, J. , Singh, A. , & Rinsch, C. (2019). The mitophagy activator urolithin A is safe and induces a molecular signature of improved mitochondrial and cellular health in humans. Nature Metabolism, 1(6), 595–603. 10.1038/s42255-019-0073-4 32694802

[acel13487-bib-0003] Brown, P. J. , Brennan, N. , Ciarleglio, A. , Chen, C. , Garcia, C. M. , Gomez, S. , Roose, S. P. , Rutherford, B. R. , Simonsick, E. M. , Spencer, R. G. , & Ferrucci, L. (2019). Declining skeletal muscle mitochondrial function associated with increased risk of depression in later life. American Journal of Geriatric Psychiatry, 27(9), 963–971. 10.1016/j.jagp.2019.03.022 PMC7388241 31104966

[acel13487-bib-0004] Choi, S. , Reiter, D. A. , Shardell, M. , Simonsick, E. M. , Studenski, S. , Spencer, R. G. , Fishbein, K. W. , & Ferrucci, L. (2016). 31P magnetic resonance spectroscopy assessment of muscle bioenergetics as a predictor of gait speed in the baltimore longitudinal study of aging. Journals of Gerontology. Series A, Biological Sciences and Medical Sciences, 71(12), 1638–1645. 10.1093/gerona/glw059 PMC5106855 27075894

[acel13487-bib-0005] Coen, P. M. , Jubrias, S. A. , Distefano, G. , Amati, F. , Mackey, D. C. , Glynn, N. W. , Manini, T. M. , Wohlgemuth, S. E. , Leeuwenburgh, C. , Cummings, S. R. , Newman, A. B. , Ferrucci, L. , Toledo, F. G. , Shankland, E. , Conley, K. E. , & Goodpaster, B. H. (2013). Skeletal muscle mitochondrial energetics are associated with maximal aerobic capacity and walking speed in older adults. Journals of Gerontology. Series A, Biological Sciences and Medical Sciences, 68(4), 447–455. 10.1093/gerona/gls196 PMC3593613 23051977

[acel13487-bib-0006] Conley, K. E. , Jubrias, S. A. , & Esselman, P. C. (2000). Oxidative capacity and ageing in human muscle. Journal of Physiology, 526(Pt 1), 203–210. 10.1111/j.1469-7793.2000.t01-1-00203.x PMC2269983 10878112

[acel13487-bib-0007] Cummings, S. R. , Studenski, S. , & Ferrucci, L. (2014). A diagnosis of dismobility–giving mobility clinical visibility: a mobility working group recommendation. JAMA, 311(20), 2061–2062. 10.1001/jama.2014.3033 24763978 PMC5012417

[acel13487-bib-0008] Ding, J. , Sidore, C. , Butler, T. J. , Wing, M. K. , Qian, Y. , Meirelles, O. , Busonero, F. , Tsoi, L. C. , Maschio, A. , Angius, A. , Kang, H. M. , Nagaraja, R. , Cucca, F. , Abecasis, G. R. , & Schlessinger, D. (2015). Assessing mitochondrial DNA variation and copy number in lymphocytes of ~2,000 sardinians using tailored sequencing analysis tools. PLoS Genetics, 11(7), e1005306. 10.1371/journal.pgen.1005306 26172475 PMC4501845

[acel13487-bib-0009] Filograna, R. , Mennuni, M. , Alsina, D. , & Larsson, N. G. (2020). Mitochondrial DNA copy number in human disease: the more the better? FEBS Letters, 595(8), 976–1002. 10.1002/1873-3468.14021 33314045 PMC8247411

[acel13487-bib-0010] Gonzalez‐Freire, M. , de Cabo, R. , Bernier, M. , Sollott, S. J. , Fabbri, E. , Navas, P. , & Ferrucci, L. (2015). Reconsidering the role of mitochondria in aging. Journals of Gerontology. Series A, Biological Sciences and Medical Sciences, 70(11), 1334–1342. 10.1093/gerona/glv070 PMC4612387 25995290

[acel13487-bib-0011] Gonzalez‐Freire, M. , Moore, A. Z. , Peterson, C. A. , Kosmac, K. , McDermott, M. M. , Sufit, R. L. , Guralnik, J. M. , Polonsky, T. , Tian, L. , Kibbe, M. R. , Criqui, M. H. , Li, L. , Leeuwenburgh, C. , & Ferrucci, L. (2020). Associations of peripheral artery disease with calf skeletal muscle mitochondrial DNA heteroplasmy. Journal of the American Heart Association, 9(7), e015197. 10.1161/JAHA.119.015197 32200714 PMC7428597

[acel13487-bib-0012] Kasashima, K. , Sumitani, M. , & Endo, H. (2012). Maintenance of mitochondrial genome distribution by mitochondrial AAA+ protein ClpX. Experimental Cell Research, 318(18), 2335–2343. 10.1016/j.yexcr.2012.07.012 22841477

[acel13487-bib-0013] Lopez‐Otin, C. , Blasco, M. A. , Partridge, L. , Serrano, M. , & Kroemer, G. (2013). The hallmarks of aging. Cell, 153(6), 1194–1217. 10.1016/j.cell.2013.05.039 23746838 PMC3836174

[acel13487-bib-0014] Moore, A. Z. , Ding, J. , Tuke, M. A. , Wood, A. R. , Bandinelli, S. , Frayling, T. M. , & Ferrucci, L. (2018). Influence of cell distribution and diabetes status on the association between mitochondrial DNA copy number and aging phenotypes in the InCHIANTI study. Aging Cell, 17(1), e12683. 10.1111/acel.12683 PMC5770782 29047204

[acel13487-bib-0015] Qian, W. , & Van Houten, B. (2010). Alterations in bioenergetics due to changes in mitochondrial DNA copy number. Methods, 51(4), 452–457. 10.1016/j.ymeth.2010.03.006 20347038

[acel13487-bib-0016] Qian, Y. , Butler, T. J. , Opsahl‐Ong, K. , Giroux, N. S. , Sidore, C. , Nagaraja, R. , Cucca, F. , Ferrucci, L. , Abecasis, G. R. , Schlessinger, D. , & Ding, J. (2017). fastMitoCalc: An ultra‐fast program to estimate mitochondrial DNA copy number from whole‐genome sequences. Bioinformatics, 33(9), 1399–1401. 10.1093/bioinformatics/btw835 28453676 PMC5860513

[acel13487-bib-0017] Short, K. R. , Bigelow, M. L. , Kahl, J. , Singh, R. , Coenen‐Schimke, J. , Raghavakaimal, S. , & Nair, K. S. (2005). Decline in skeletal muscle mitochondrial function with aging in humans. Proceedings of the National Academy of Sciences of the United States of America, 102(15), 5618–5623. 10.1073/pnas.0501559102 15800038 PMC556267

[acel13487-bib-0018] Zampino, M. , Semba, R. D. , Adelnia, F. , Spencer, R. G. , Fishbein, K. W. , Schrack, J. A. , Simonsick, E. M. , & Ferrucci, L. (2020). Greater skeletal muscle oxidative capacity is associated with higher resting metabolic rate: Results from the baltimore longitudinal study of aging. Journals of Gerontology. Series A, Biological Sciences and Medical Sciences, 75(12), 2262–2268. 10.1093/gerona/glaa071 PMC7751004 32201887

