# Supplementary material for: Mitochondrial DNA copy number and heteroplasmy load correlate with skeletal muscle oxidative capacity by P31 MR spectroscopy
Source: Aging Cell. 2021 Oct 6;20(11):e13487. doi: 10.1111/acel.13487 (PMC8590093; doi:10.1111/acel.13487)
Supplement: Supplementary file 1 — Supplementary Material [file ACEL-20-e13487-s001.docx]

**Supporting Information**

**Experimental procedures**

**Study population**

We identified 230 participants from the Baltimore Longitudinal Study of Aging (BLSA) with concurrent measurements of mitochondrial DNA copy number (mtDNA-CN) and heteroplasmy load as well as muscle oxidative capacity assessed using ^31^P MRS between 2013 and 2018. The BLSA is a longitudinal study with continuous enrollment that began in 1958 (Shock et al. 1984, Ferrucci 2008). The National Institutes of Health Institutional Review Board approved the study protocol. All participants provided written informed consent.

**Mitochondrial DNA copy number and heteroplasmy load**

Both mtDNA-CN and mtDNA heteroplasmy load were estimated based upon data from standard whole-genome sequence (WGS) of BLSA participants; after buffy coat DNA extraction, sequencing was performed on a HiSeq X Ten sequencer (version 2.5 chemistry, Illumina) that generated 150 base-long pair-end sequencing reads; the resulting average sequencing coverage for nuclear genome was 35X, while the average coverage of the mtDNA was 3,499x. All alleles of the called genotype at heteroplasmic positions were observed at least once in both forward and reverse strand sequence reads, and the minor allele fraction was ≥ 3%.

**Mitochondrial energetics**

*In vivo* ^31^P-MRS measurements of the concentrations of the phosphorus-containing metabolites phosphocreatine (PCr), inorganic phosphate (Pi), and ATP were obtained from the vastus lateralis muscle using ^31^P MRS at 3T, following a standardized protocol (Choi et al. 2016). Briefly, a series of pulse-acquire ^31^P spectra were obtained before, during, and after a ballistic knee extension exercise of average duration 30 seconds, using a 10-cm ^31^P-tuned surface coil (PulseTeq, UK) fastened above the left thigh. Signals were averaged over four successive acquisitions for signal-to-noise ratio enhancement, to obtain a total of 75 spectra. Exercise duration was optimized through requiring a depletion in PCr spectral peak height of 50% to 67% relative to its initial baseline values. Spectra were processed with jMRUI software (MRUI Consortium, v5.2), and metabolite concentrations were calculated by nonlinear least-squares fitting implemented through AMARES (Naressi et al. 2001).

**Other measures of interest**

Demographic information included age, sex, and body mass index. Physical function was measured as gait speed at a usual pace over 6 meters. Health-related conditions included cancer, non-skin cancer, and diabetes by self-report. Smoking status was defined as non-smokers versus current or former smokers by self-report. Physical activity was measured using a standardized questionnaire. Based on minutes of moderate to vigorous physical activity per week, participants were categorized into low (≤30 minutes), median (31-149 minutes), and high physical activity groups (≥150 minutes) (Taylor et al. 1978, Brach et al. 2004).

**Statistical analysis**

The association of mtDNA-CN or heteroplasmy load with τ_PCr_ was tested using multivariable linear regression (Table S1. Model 1 and Model 2), adjusted for age, sex, extent of PCr depletion, autosomal sequencing coverage, total white blood cell count, percentages of white blood cell subtypes, and platelet count (SAS v9.4; SAS Institute, Inc, Cary, NC). These covariates were maintained in multivariate regression based on biological/technical rationales and/or relationships with mtDNA parameters or τPCr that have been described in other populations.

We further tested the interaction between mtDNA-CN and heteroplasmy load by adding an interaction term in the model (i.e. Table S1. Model 3). Sensitivity analyses were also performed by excluding individuals with diabetes and non-skin cancer.

**Supplementary Tables**

**Table S1: Associations of mitochondrial DNA copy number and heteroplasmy load with** τ_PCr_ **(n=230)**

|  |  | Basic adjustment | Full adjustment |
| --- | --- | --- | --- |
|  |  | **β (SE), p-value** | |
| Model 1 | **mtDNA-CN** | -0.135 (0.077)  0.082 | -0.119 (0.083)  0.153 |
| Model 2 | **Heteroplasmy load** | -0.113 (0.064)  0.078 | - |
| Model 3 | **mtDNA-CN** | -0.137 (0.076)  0.074 | -0.117 (0.082)  0.154 |
|  | **Heteroplasmy load** | -0.068 (0.065)  0.301 | -0.070 (0.066)  0.290 |
|  | **mtDNA-CN * heteroplasmy load interaction** | -0.166 (0.075)  0.026 | -0.159 (0.076)  0.037 |

Footnote. mtDNA-CN=mitochondrial DNA copy number. Values of mtDNA-CN, heteroplasmy load, and τ_PCr_ were computed as standardized Z scores. In model 1 and model 3: Basic adjustment includes age, sex, extent of PCr depletion, autosomal sequencing coverage, total white blood cell count, and platelet count. Full adjustment includes covariates in the basic adjustment, along with white blood cell subsets (% neutrophils, % lymphocytes, % monocytes, % eosinophils, and % basophils). In model 2, basic adjustment includes age, sex, extent of PCr depletion, and autosomal sequencing coverage.

**Table S2: Associations of mitochondrial DNA copy number with** τ_PCr_ **stratified by a median split of heteroplasmy load at a value of 3**

|  | Heteroplasmy load ≤ 3  (n=146) | Heteroplasmy load > 3  (n=84) |
| --- | --- | --- |
|  | **β (SE)**  **p-value** | |
| Model 1: basic adjustment | 0.040 (0.113)  0.721 | -0.311 (0.104)  0.003 |
| Model 2: full adjustment | 0.046 (0.119)  0.702 | -0.236 (0.115)  0.044 |

Footnote. In model 1, basic adjustment includes age, sex, extent of PCr depletion, autosomal sequencing coverage, total white blood cell count, and platelet count. In model 2, full adjustment adds subsets of the white blood cell count (% neutrophils, % lymphocytes, % monocytes, % eosinophils, and % basophils).

**Table S3: Sex-stratified associations of mitochondrial DNA copy number with** τ_PCr_ **stratified by a median split of heteroplasmy load at a value of 3**

|  |  | Heteroplasmy load ≤ 3 | Heteroplasmy load > 3 |
| --- | --- | --- | --- |
|  |  | **β (SE)**  **p-value** | |
| Men |  | **(n=66)** | **(n=43)** |
|  | Model 1: basic adjustment | -0.055 (0.172)  0.748 | -0.477 (0.148)  0.003 |
|  | Model 2: full adjustment | -0.155 (0.258)  0.552 | -0.356 (0.190)  0.071 |
| Women |  | **(n=80)** | **(n=41)** |
|  | Model 1: basic adjustment | 0.101 (0.118)  0.395 | -0.262 (0.131)  0.053 |
|  | Model 2: full adjustment | 0.112 (0.141)  0.433 | -0.254 (0.183)  0.176 |

Footnote. In model 1, basic adjustment includes age, extent of PCr depletion, autosomal sequencing coverage, total white blood cell count, and platelet count. In model 2, full adjustment adds subsets of the white blood cell count (% neutrophils, % lymphocytes, % monocytes, % eosinophils, and % basophils).


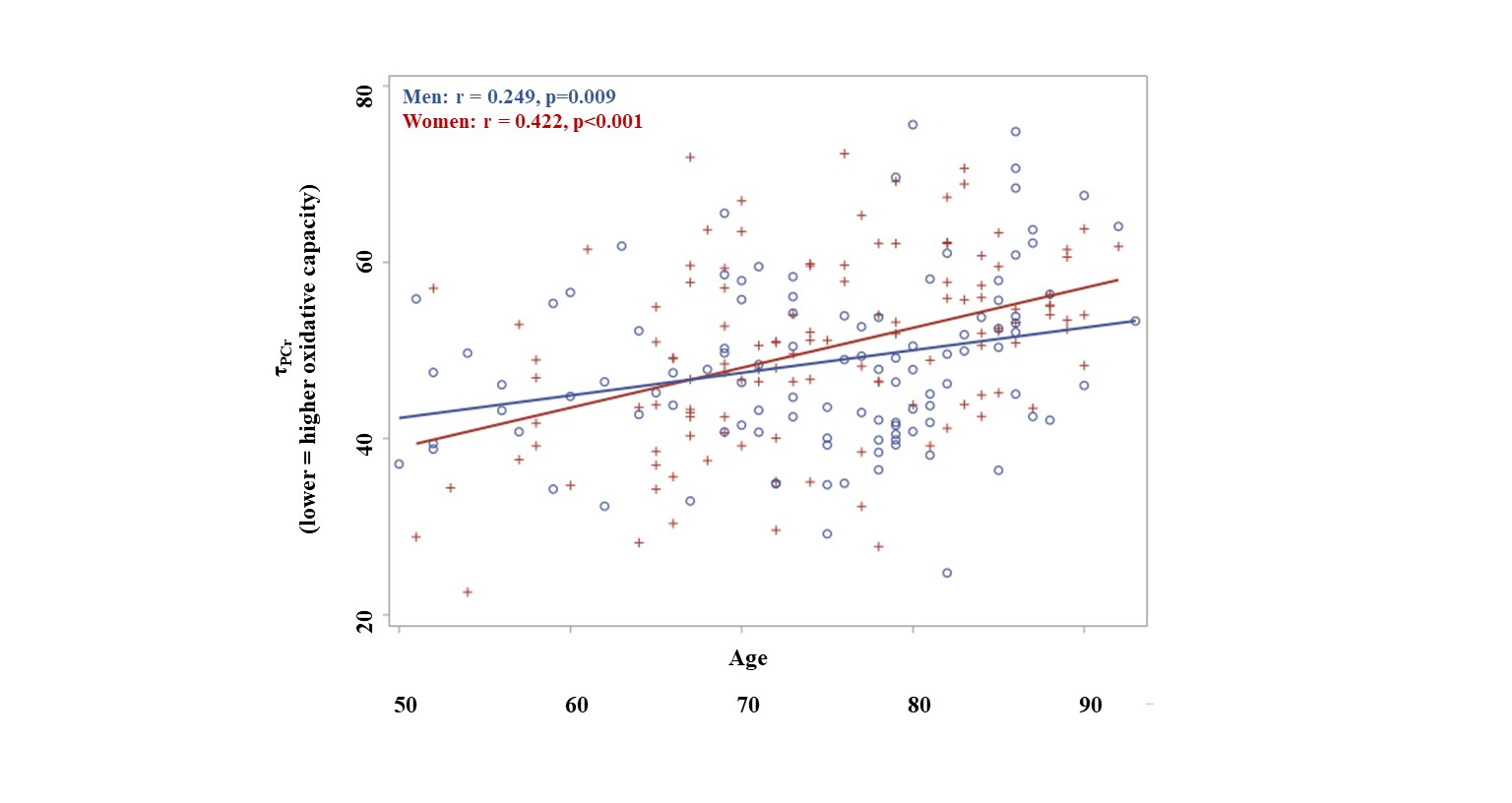


**Figure S1. Scatter plots of τ_PCr_ as a function of age**

**References:**

Brach, J. S., et al. (2004). "The association between physical function and lifestyle activity and exercise in the health, aging and body composition study." J Am Geriatr Soc **52**(4): 502-509.

Choi, S., et al. (2016). "31P Magnetic Resonance Spectroscopy Assessment of Muscle Bioenergetics as a Predictor of Gait Speed in the Baltimore Longitudinal Study of Aging." J Gerontol A Biol Sci Med Sci **71**(12): 1638-1645.

Ferrucci, L. (2008). "The Baltimore Longitudinal Study of Aging (BLSA): a 50-year-long journey and plans for the future." J Gerontol A Biol Sci Med Sci **63**(12): 1416-1419.

Naressi, A., et al. (2001). "Java-based graphical user interface for MRUI, a software package for quantitation of in vivo/medical magnetic resonance spectroscopy signals." Comput Biol Med **31**(4): 269-286.

Shock, N. W., et al. (1984). Normal Human Aging: The Baltimore Longitudinal Study of Aging. Washington, D.C., National Institutes of Health.

Taylor, H. L., et al. (1978). "A questionnaire for the assessment of leisure time physical activities." J Chronic Dis **31**(12): 741-755.
